# Color and Spatial Frequency Provide Functional Signatures of Retinotopic Visual Areas

**DOI:** 10.1523/JNEUROSCI.1673-23.2024

**Published:** 2024-11-04

**Authors:** Spencer R. Loggia, Stuart J. Duffield, Kurt Braunlich, Bevil R. Conway

**Affiliations:** ^1^National Eye Institute, Bethesda, Maryland 20892; ^2^Department of Neuroscience, Brown University, Providence, Rhode Island; ^3^National Institute of Mental Health, Bethesda, Maryland 20892

**Keywords:** color, cortical organization, fMRI, macaque, retinotopy, vision

## Abstract

Primate vision relies on retinotopically organized cortical parcels defined by representations of hemifield (upper vs lower visual field), eccentricity (fovea vs periphery), and area (V1, V2, V3, V4). Here we test for functional signatures of these organizing principles. We used functional magnetic resonance imaging to measure responses to gratings varying in spatial frequency, color, and saturation across retinotopically defined parcels in two macaque monkeys, and we developed a Sparse Supervised Embedding (SSE) analysis to identify stimulus features that best distinguish cortical parcels from each other. Constraining the SSE model to distinguish just eccentricity representations of the voxels revealed the expected variation of spatial frequency and S-cone modulation with eccentricity. Constraining the model according to the dorsal/ventral location and retinotopic area of each voxel provided unexpected functional signatures, which we investigated further with standard univariate analyses. Posterior parcels (V1) were distinguished from anterior parcels (V4) by differential responses to chromatic and luminance contrast, especially of low-spatial-frequency gratings. Meanwhile, ventral parcels were distinguished from dorsal parcels by differential responses to chromatic and luminance contrast, especially of colors that modulate all three cone types. The dorsal/ventral asymmetry not only resembled differences between candidate dorsal and ventral subdivisions of human V4 but also extended to include all retinotopic visual areas, starting in V1 and increasing from V1 to V4. The results provide insight into the functional roles of different retinotopic areas and demonstrate the utility of SSE as a data-driven tool for generating hypotheses about cortical function and behavior.

## Significance Statement

This study demonstrates a new analysis, Sparse Supervised Embedding (SSE), which promises to be useful for visualizing and understanding complex neuroimaging datasets. The paper uses SSE to explore the functional roles of retinotopic visual areas (V1, V2, V3, V4, V3a, MT). The results show that retinotopic areas parcellated by representations for eccentricity and upper/lower visual hemifield have functional signatures, which are defined by unique combinations of responses to color, spatial frequency, and contrast. The functional signatures provide hypotheses for the different roles that the parcels play in vision and help resolve apparent differences between human and macaque visual cortex organization.

## Introduction

The computations performed by each retinotopically organized visual area in the putative visual-processing hierarchy (V1, V2, V3, V4) remain unclear. The retinotopic organization of these areas leaves an imprint on higher-order areas (Hasson et al., 2003; Lafer-Sousa and Conway, 2013; Conway, 2018; Arcaro et al., 2017; Klink et al., 2021) and influences visual behavior. For instance, acuity is determined by a target's location relative to the fovea, and perception varies with polar angle (Carrasco and McElree, 2001; Levine and McAnany, 2005; Quek and Finkbeiner, 2015; Himmelberg et al., 2023). Such perceptual asymmetries are not fully explained by retinal factors, pointing to cortical explanations (Kupers et al., 2022), but the distinct roles of different retinotopic areas remain elusive. One idea is that the sequence of retinotopic areas builds complex receptive fields (Freeman et al., 2013; Vernon et al., 2016; Ponce et al., 2019). According to this idea, the visual areas, either singly or in clusters, represent computational units (Wandell et al., 2005; Winawer and Witthoft, 2015), but there are other possibilities. For example, multiple areas could reflect selective pressure to smoothly represent the visual field at multiple scales (Tarhan et al., 2021). Determining the roles of retinotopic areas would be facilitated by quantitative data on cortical responses in macaque monkey, a standard model of the human visual system.

Here we ask: To what extent can combinations of simple stimulus features distinguish retinotopically defined parcels? Our objectives are twofold. First, to advance a quantitative framework for understanding neural responses across the visual-processing hierarchy and their connection to perception, and second, to facilitate linking neural activity in monkeys to psychophysical and imaging data in humans. Achieving these objectives will allow tests of evolutionary homologies and help reveal the extent to which the visual systems are similar in macaque and human.

We first used functional magnetic resonance imaging (fMRI) to parcellate the macaque visual cortex by eccentricity, upper and lower visual field, and visual area. We then used fMRI to measure responses to gratings varying in color, spatial frequency, and cone contrast, as done in human subjects (Wade et al., 2002; Brewer et al., 2005; Liu and Wandell, 2005; Mullen, 2019). We analyze the data with a new multivariate analysis method we call Sparse Supervised Embedding (SSE) that discovers differences between subdivisions of a dataset in a high-dimensional space. Similar methods such as linear discriminant analysis (LDA) or linear classifiers have been used to separate stimulus-defined classes by voxel responses (Cox and Savoy, 2003; Pardo et al., 2006; Lin et al., 2021). SSE flips this logic, instead uncovering sparse combinations of stimuli that best distinguish cortical parcels. The results provide a functional signature for the cortical parcels, somewhat analogous to voxel decomposition (Norman-Haignere, 2015).

Unlike traditional univariate analyses, which report response magnitude to individual stimuli, SSE uncovers structure determined by the relative responses to all stimuli in a dataset. SSE is well suited to our purpose because rather than simply classifying parcels based on the average response of voxels in each parcel, it finds a space that yields high variance between these average responses and low variance of the voxel responses within each parcel. The axes of this space are those that best separate the cortical parcels and are interpretable as linear combinations of features—in our case, gratings varying in color, contrast, and spatial frequency. The organization of the parcels along the axes, to the extent there is any organization, will be informative about the functional relationships of the parcels. In the present work, SSE discovered an axis that organizes the parcels by eccentricity, and the axis features are those known to vary with eccentricity (Wright and Johnston, 1983; Curcio et al., 1991; Vanston and Crognale, 2018; Broderick et al., 2022), which validates the approach. SSE also produced axes that separate parcels by upper and lower visual field and by location along the putative cortical processing hierarchy, discovering functional signatures of retinotopic visual areas and their upper and lower visual field representations.

## Materials and Methods

### fMRI acquisition

Imaging acquisition was the same as in Lafer-Sousa et al. (2012) and Lafer-Sousa and Conway (2013). The V1 data analyzed presently contributed to those two reports, but none of the data on responses in V2, V3, V4, MT, or V3a presented here have previously been published; all the data analyzed in the present report are available in open access (https://openneuro.org/datasets/ds005521). Two alert rhesus macaques (7–8 kg, M1 and M2) were scanned at the Martinos Imaging Center at Massachusetts General Hospital in a 3 Tesla Allegra scanner (Siemens) using a custom-made four–channel send–receive surface coil (Athinoula A. Martinos Center for Biomedical Imaging). Images were acquired using standard echoplanar imaging methods with 2 s repetition time (TR), each repetition acquiring a 98 × 63 × 98 voxel matrix with 1 mm isotropic voxels. Animals were trained using juice rewards to sit in a sphinx position in a custom-built plastic chair placed inside the bore of the horizontal scanner while fixating a central target on a display screen. Animals were required to fixate throughout the experiment to receive reward. Head position was maintained with custom plastic head posts that were surgically implanted (see below, Surgical procedures; Lafer-Sousa et al., 2012). Eye movements were tracked with an infrared eye tracker (ISCAN). Animals were rewarded for maintaining fixation within a degree of the central fixation target. Monocrystalline iron oxide nanoparticle (MION; AMAG Pharmaceuticals; 8–10 mg/kg, diluted in approximately equal volume of saline) was injected intravenously in the saphenous vein immediately prior to scanning to improve the magnetic resonance signal (Vanduffel et al., 2001). High-resolution anatomical scans (0.35 mm × 0.35 mm × 0.35 mm in M1 and 0.35 mm × 0.4 mm × 0.35 mm in M2) were obtained while the animals were lightly sedated during a separate scanning session. All imaging and surgical procedures follow local and National Institutes of Health guidelines and were approved by the Harvard Medical School Institutional Animal Care and Use Committee.

### Experimental design and statistical analysis

**Figure 1.**
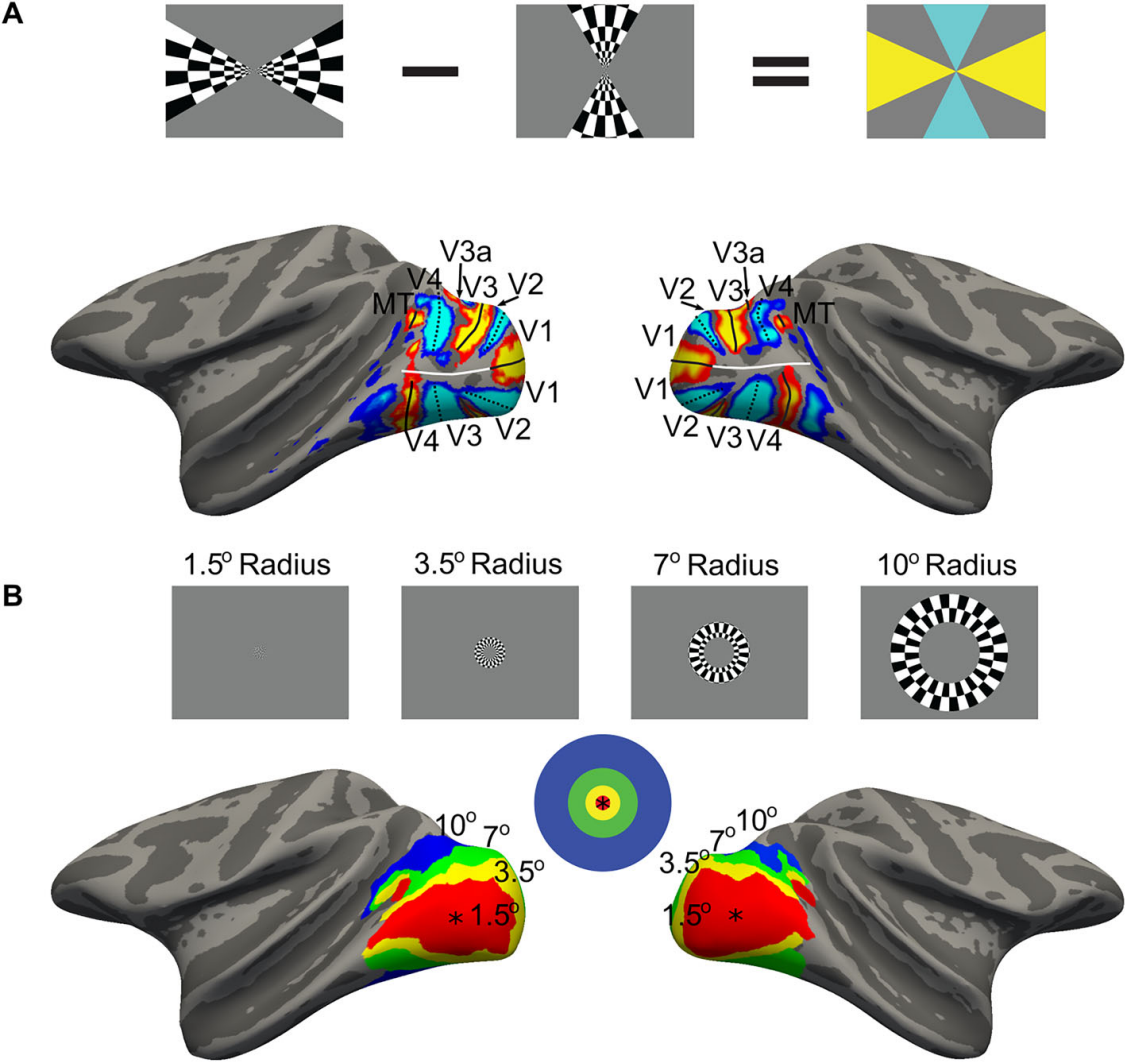
Parcellation of the retinotopic visual cortex in macaque monkey. ***A***, Individual-specific functional parcellation. To delineate retinotopic areas in each macaque, using a block design, the animals were shown checkerboard patterns along the vertical and horizontal meridians (icon). The panel shows the contrast maps between these two conditions displayed on the inflated surface of Monkey 1 (M1); responses biased for the vertical meridian (cool colors) and horizontal meridian (warm colors). Each retinotopic area is bounded by peak activation along the horizontal meridian (solid black line) and vertical meridian (dotted black line). The most posterior horizontal meridian delineates dorsal V1 (representing the lower visual field) from ventral V1 (representing the upper visual field). The most posterior vertical meridian representation separates V1 from V2. Progressing from posterior to anterior, V2 is then separated from V3 by the next horizontal meridian representation, and V3 is separated from V4 by the next vertical meridian representation. V4 is bounded anteriorly by a fragmented horizontal meridian representation. V3a is not clearly visible in the lateral projection shown in the figure (it is situated on the dorsal surface), but it is contained by its own meridian representations. The solid white line extending anteriorly from the horizontal meridian representation in V1 shows the separation of dorsal and ventral parcels that tracks through the foveal confluence. ***B***, To define voxels by eccentricity responses, checkerboard rings and circles were presented in a blocked paradigm, and the statistical contrasts between these conditions were used to create parcels encompassing the central 1.5° and nested annuli extending to 3.5°, 7°, and 10° (icon). The eccentricity parcellation is shown for M1. Note the existence of two representations of the fovea; the larger one corresponds to the confluence of the V1–V4 cluster and the other corresponds to the MT cluster.

**Figure 2.**
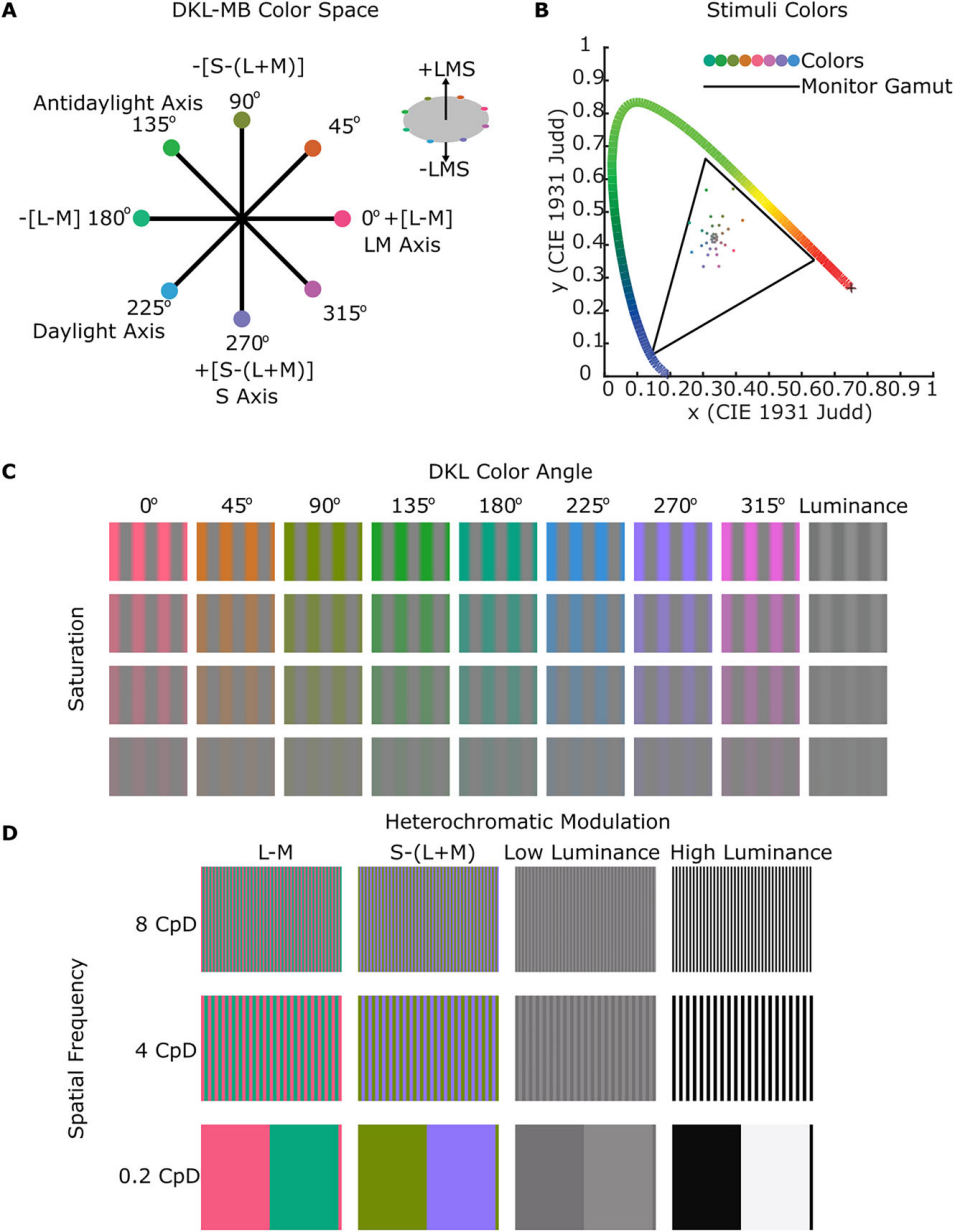
Overview of the stimuli used to identify the functional signatures for retinotopic parcels. ***A***, The colors of the stimuli were defined in a cone-opponent color space. An equiluminant plane through the space is shown. Colors included those of the two cardinal cone-opponent axes, the two intermediate axes, and the luminance axis (orthogonal to the equiluminant plane, inset). These axes are labeled LM (0 and 180°), daylight (45 and 225°), S (90 and 270°), antidaylight (135 and 315°), and luminance (LMS). ***B***, CIE-1931 chromaticity diagram showing the various saturation levels of the stimuli colors used in one set of experiments (Experiment 3). The continuous color horseshoe shows where the spectrum plots in the chromaticity space, while the dots show the stimuli and the asterisk indicates the chromaticity of the adapting gray background. See Table 1 for the cone contrasts of the stimuli. ***C***, The set of color-gray gratings used in Experiment 3, varying in hue angle (columns) and saturation (rows). The spatial frequency of the gratings was 0.5 CpD. ***D***, The set of heterochromatic gratings used in Experiment 4, varying in axis through the color space (columns) and spatial frequency (rows, CpD). See Table 2 for the cone contrasts of the stimuli.

Four fMRI experiments were conducted over the course of seven sessions in M1 and six sessions in M2. In Experiment 1, we measured responses to vertical and horizontal flickering checkerboard wedges to define retinotopic areas (Fig. 1*A*). In Experiment 2, we measured responses to checkerboard patterns restricted to rings of different eccentricity (Fig. 1*B*). In Experiment 3, we measured responses to color-gray gratings in which the colors were defined by a cone-opponent color space (Fig. 2*A*,*B*; MacLeod and Boynton, 1979; Derrington et al., 1984); the stimuli varied in hue and saturation (Fig. 2*C*). Finally, in Experiment 4, we measured responses to heterochromatic gratings also defined by the cone-opponent color space but varying in spatial frequency (Fig. 2*D*). Each scan session consisted of 13–24 stimulus runs, details of which are described below.

The color space is defined by two equiluminant cone-opponent axes, one of which consists of colors that modulate responses of the L and M cones without changing the activity of the S cones (the L-M axis, demarcated in Fig. 2*A* by color angles 0 and 180), and the other consists of colors that modulate responses of S cones without changing the activity of the L or M cones (demarcated by color angles 90 and 270). The space has an orthogonal luminance axis that is modulated by the change in L, M, and S cones (Fig. 2*A*, inset). Colors in each experiment selectively modulated the L-M axis, the S axis, the luminance axis, or two intermediate axes of the equiluminant plane that modulate all three cone types. The colors of the two intermediate axes correspond roughly to the daylight locus (orange/blue; 45 and 225°) and the antidaylight locus (green/magenta; 135 and 315°). We refer to these five chromatic dimensions as LM, S, LMS, daylight, and antidaylight, following others (Goddard et al., 2010; Lafer-Sousa et al., 2012). The gamut of the color space was bounded by the monitor gamut (Fig. 2*B*, triangle).

The stimuli were presented on a screen 48.2 cm away from the animal, with a JVC DKLA projector (1,024 × 768 pixel resolution). For Experiments 1, 3, and 4, the projected image subtended 22.5 × 16.9 cm (27 × 20 degrees of visual angle, DvA), and for Experiment 2, the projected image subtended 35 × 26.8 cm (41 × 31 DvA). The projector's luminance output was linearized, and colors were measured and calibrated using spectral readings taken from a spectroradiometer (PR-655, Photo Research). Spectra were multiplied by the Judd-revised CIE 1931 color matching functions to derive CIE *xy* coordinates (Fig. 2*B*). All stimuli were presented in a blocked paradigm. Stimulus blocks were interleaved with adapting-gray blocks.

The first experiment (Experiment 1, Fig. 1*A*) allowed us to identify horizontal and vertical meridian representations which define the boundaries between retinotopic visual areas (for review, see Vanduffel et al., 2014). Stimuli consisted of black-and-white checkerboard wedges that flickered between complementary checkerboard patterns every 1 s at 99% luminance contrast. The stimuli were two wedges that radiated from the central fixation spot. In one block, the wedges spanned the vertical meridian (60° wedge); in the other block, the wedges spanned the horizontal meridian (30° wedge). Blocks were 32 s (16 TRs), and each run had 16 blocks. For all runs, the conditions were ordered horizontal, gray, vertical, and gray, repeated four times. A total of 13 runs of this experiment were collected in M1, and 14 runs were collected in M2.

The second experiment was an eccentricity mapping experiment (Experiment 2, Fig. 1*B*). Stimuli consisted of LMS, LM, or S checkerboards presented in rings centered on the fixation point; the checkerboards flickered between complementary checkerboard patterns every 1 s. Rings extended from the central fixation spot to a width of 20 DvA, with each successively larger ring having an inner radius equal to the outer radius of the previous ring. Rings had outer radii of 1.5°, 3.5°, 7°, and 10°. Blocks showing one of the rings were interleaved with blocks of the adapting-gray background. Blocks were 32 s (16 TRs), and each run had 17 blocks. There were three run orders, with only the colors of the checkboards varying across the orders. For all three run orders, the gray blocks and rings proceeded as gray, 1.5°; gray, 3.5°; gray, 7°; and gray, 10°, repeated twice, with a final gray block. For the first order, the first four ring blocks were S checkerboards, and the second four ring blocks were LM; the second order had S followed by LMS checkerboards; the third order had LMS followed by LM checkerboards. A total of 24 runs were collected in M1 and 21 runs were collected in M2.

In the third experiment, we presented color-gray “trapezoidal” gratings with a spatial frequency of 0.5 cycles per degree (CpD; Fig. 2*C*). The structure of the gratings is trapezoidal when plotting the cone contrast of the colors along the line perpendicular to the gratings. The saturated or fully gray portions of the gratings made up 80% of each cycle; the transition between the fully gray and fully saturated portions of the gratings was progressive such that the cone contrast from one side of the grating to the other was linear. This spatial structure helps mitigate chromatic aberration. The gratings were presented in nine hues, defined by the eight poles of the four axes in the equiluminant color plane and the luminance axis. Each hue was presented at four saturation levels (10, 30, 50, or 95% of the maximum saturation of the display for the colored gratings; 1, 2.5, 5.5, or 8.7% luminance contrast for the achromatic gratings; contrasts were computed as Michelson contrast; Table 1). In total, there were 32 colored grating blocks and 4 achromatic grating blocks. The gratings drifted horizontally at a speed of 0.8 cycles per second, and the direction of the drift changed every 2 s. In each run, blocks of gratings were interleaved with blocks of gray. The blocks were 28 s (14 TRs), and each run had 19 blocks (10 blocks of gray and 9 blocks of gratings). The full cycle of 36 gratings was presented over four runs. Each of the four runs presented 8 of the 32 colored gratings, plus one of the 4 achromatic gratings (the eight gratings in each run were unique, i.e., not shown in any of the other three runs). Each block within a run was a unique color, randomly selected from one of the four saturation levels in such a way that all hues were presented in all runs. For each run, the hues were presented in one of the four counterbalanced sequences, and the achromatic grating was shown in the middle of the sequence. This stimulus paradigm structure helps ensure that the intervening gray blocks provide a baseline reference throughout the experiment. A total of 49 runs were collected in M1, and 58 runs were collected in M2. Each block type was presented between 26 and 28 times.

**Table 1.** Michelson cone contrasts for L, M, and S channels for Experiment 3 gratings stimuli

Color	Sat. level	%L	%M	%S
DKL 0	95	5.730	−10.198	0.066
DKL 45	95	4.356	−7.987	−40.341
DKL 90	95	−0.078	−0.817	−59.771
DKL 135	95	−3.898	7.641	−43.922
DKL 180	95	−5.361	11.012	0.303
DKL 225	95	−4.073	7.226	42.951
DKL 270	95	−0.398	−0.408	60.353
DKL 315	95	3.745	−7.725	42.740
Light	95	8.400	8.325	5.795
Dark	95	−8.989	−8.901	−6.309
DKL 0	50	3.523	−4.981	0.253
DKL 45	50	2.431	−3.558	−22.835
DKL 90	50	0.353	0.268	−32.172
DKL 135	50	−1.725	4.252	−22.838
DKL 180	50	−2.627	5.839	0.347
DKL 225	50	−2.322	3.686	22.322
DKL 270	50	−0.361	−0.426	31.988
DKL 315	50	1.830	−4.139	22.837
Light	50	5.678	5.638	4.008
Dark	50	−5.298	−5.223	−3.808
DKL 0	30	2.189	−3.165	0.314
DKL 45	30	1.615	−2.080	−13.190
DKL 90	30	0.233	0.220	−19.798
DKL 135	30	−1.253	2.212	−14.045
DKL 180	30	−1.436	3.799	−0.047
DKL 225	30	−1.529	2.087	13.540
DKL 270	30	−0.036	−0.125	19.624
DKL 315	30	1.197	−2.642	13.868
Light	30	2.520	2.516	1.741
Dark	30	−2.526	−2.510	−1.757
DKL 0	10	0.674	−1.177	0.239
DKL 45	10	0.733	−0.514	−3.895
DKL 90	10	−0.264	−0.419	−6.746
DKL 135	10	−0.309	0.970	−4.477
DKL 180	10	−0.720	1.056	−0.705
DKL 225	10	−0.341	0.848	3.940
DKL 270	10	−0.058	−0.033	6.443
DKL 315	10	0.592	−0.739	4.771
Light	10	1.219	1.283	0.889
Dark	10	−1.071	−1.072	−0.932

The fourth experiment presented heterochromatic gratings with a spatial frequency of either 0.2, 4, or 8 CpD. The colors of the two phases of a given grating were defined by the poles of LM axis, the S axis, or the luminance axis (Fig. 2*D*). The colors of the colored gratings were set at 90% of the saturation of the display defined in the DKL-MB color space; the achromatic gratings were of either 90 or 9% luminance contrast (see Table 2 for cone contrasts). Blocks of gratings were interleaved with blocks of gray. During a given block, the grating was stationary and alternated in counter-phase between complementary patterns every 1 s. The blocks were 32 s (16 TRs), and each run had 25 blocks, which included all 12 gratings conditions and the 13 intervening gray blocks. The stimulus blocks were shown in a counterbalanced sequence across runs. A total of 14 runs were collected in M1, and 13 runs were collected in M2. Each block type was presented 27 times.

**Table 2.** Michelson cone contrasts for L, M, and S channels for Experiment 4 dichromatic gratings stimuli

Color	%L	%M	%S
DKL 0–180	6.957	12.923	0.400
DKL 90–270	0.110	0.250	71.845
Luminance	7.137	7.067	6.487

fMRI data preprocessing is described below. *F* tests for linear regressions were computed with MATLAB's *fitlm* function. Multiway ANOVAs, ANCOVAs, and subsequent Tukey's honest significant difference (HSD) tests were computed with MATLAB's *anovan*, *ancova*, and *multcompare* functions. Multiway ANCOVA was computed using the MANCOVAN toolbox (Gruner, 2010). In cases where bootstrapping was used to generate confidence intervals, data were independently resampled 1,000 times. Variance and expected value were determined by fitting a normal distribution over all resamples. *P* values and statistical tests used are presented in the figures, figure legends, and results section. The SSE analysis method is described in detail below, SSE analysis.

### fMRI preprocessing

The raw data were unpacked from DICOM (Digital Imaging and Communications) to NIfTI (Neuroimaging Informatics Technology Initiative) format using dcm2niix (Li et al., 2016). The images underwent thermal denoising using the NORDIC algorithm (Vizioli et al., 2021) to improve the signal-to-noise ratio. Images were reoriented from the sphinx position. Data were motion corrected with the FSL (fMRI-Brain Software Library) motion-correction algorithm (MCFLIRT) with 12 degrees of freedom (Smith et al., 2004). The functional volumes were then coregistered to the anatomical volumes using ITK-SNAP's linear registration tool v3.6.0 (Yushkevich et al., 2006) and ANTs nonlinear registration algorithm (Avants et al., 2011) to get the closest mapping of the functional volumes to the anatomical image. Blocks were one-hot encoded and convolved with the MION hemodynamic response function (HRF) to create design matrices (Vanduffel et al., 2001). No spatial smoothing was applied. Nilearn's general linear model module (https://nilearn.github.io/) was used to calculate *β* coefficients and create statistical contrast maps, using drift regressors up to the third-order polynomial to account for fMRI signal drift. The *β* coefficients for each block were divided by the *β* coefficients for the intervening gray blocks for each run.

### Anatomical processing and region of interest definition

To create surfaces of the macaque brain, the high-resolution anatomical images were skull stripped, white matter regions were labeled, and surfaces were generated and inflated with FreeSurfer (Smith et al., 2004). Retinotopic parcels were defined using functional data obtained in each animal (Experiment 1). Significance maps showing the contrast of responses to vertical versus horizontal were shown on the inflated surface of each animal, and the peak bias of the vertical-greater-than-horizontal responses was used to define the boundary between V1 and V2 and the boundary between V3 and V4. The peak bias of the horizontal-greater-than-vertical responses were used to define the upper and lower visual field representations of V1 and the boundary between V2 and V3. MT+ was defined such that it bordered V4d along a shared horizontal meridian and featured a continuous eccentricity map in a three quarters circle around the foveal representation in the superior temporal sulcus (Fig. 1*B*; Brewer et al., 2002; Kolster et al., 2014). Surface labels of the retinotopic areas were transformed into the anatomical volume space. The functionally defined retinotopic areas were cross-referenced with the Paxinos illustrated atlas (Paxinos et al., 2000), the D99 atlas (Reveley et al., 2016), and the CHARM atlas (Jung et al., 2021), producing retinotopic regions of interest (ROIs) for each subject.

Retinotopic areas V1, V2, V3, and V4 were subdivided into dorsal and ventral ROIs by extending the horizontal meridian of V1 through the center of the foveal confluence in each hemisphere of each subject's surface (Fig. 1*A*, white line). The surface labels were then back-projected into the anatomical space and used as a reference to divide the retinotopic ROIs into ventral and dorsal parcels, corresponding to the upper and lower visual field representations of each retinotopic area. V3a and MT+, being located on the dorsal surface of the cortex, were not subdivided, despite these areas having representations of the upper and lower visual field (Gattass et al., 1988; Kolster et al., 2014; Zhu and Vanduffel, 2019).

To determine eccentricity representations, statistical contrasts of the responses elicited by neighboring rings were generated, and voxels were assigned to eccentricities eliciting the maximum *z*-score, moving outward from foveal regions to the periphery (Fig. 1*B*). Together, the functional parcellation provides the eccentricity, visual area, and upper-versus-lower visual field representation of each voxel.

### SSE analysis

We developed a data-driven method that discovers a low-dimensional space *E* that best captures the relationships between the retinotopic parcels, *R*. We refer to this method as SSE. The goal of the method is to use the responses to the various visual stimuli to uncover functional relationships between the parcels. So, unlike other approaches that use fMRI responses to determine relationships among stimuli, we use the fMRI responses to determine relationships among the cortical parcels. The results allow us to test the hypothesis that color gratings of varying spatial frequency and contrast provide a functional signature of the parcels. Given a number of voxels *v*, a number of stimulus conditions *m*, and a number component vectors *k*, we find the *(m* × *k*) projection matrix *T* that takes the input data matrix (in this case a *v* × *m* standardized *β* coefficient matrix) into a *k*-dimensional Euclidean space *E*, such that *T* maximizes the pairwise distance between parcel centers while minimizing variance within those parcels. The final objective is the sum of the interparcels distance term, the negated intraparcel variance term, and an L1 regularization term over *T* as follows (Eq. 1):

$$\underset{T}{argmax}\;\underset{\forall f_{1}\in R}{\sum }\;\underset{\forall f_{2}\in R}{\sum }\;\frac{M_{f_{1},f_{2}}\sqrt{{\left(\bar{\beta_{f_{1}}T}-\bar{\beta_{f_{2}}T}\right)}^{2}}}{|R{|}^{2}}-\frac{1}{\left|R\right|}\underset{\forall f\in R}{\sum }\;\sum \;\;|\mathrm{cov}(\beta_{f}T)|+S\sum \;\;|T|.$$

#### Equation 1: the SSE objective function

In Equation 1, *R* is the set of ROI labels (and *|R|* is the number of ROIs), *T* is the transform we are trying to discover (the set of *k* length m component vectors), *β* is the beta matrix with *m* features, *k* is the number of target components or dimensions, and *M* is a weight matrix that allows control over the importance of separating each pair of classes. We use cov to denote computing the covariance matrix over the embedding dimensions. *S* (the sparsity term) scales the magnitude of the L1 regularization term; it is used to reduce overfitting, reduce variability between independent runs, and encourage more interpretable component vectors. For most of our analyses, *S* is set to 0.005; in a control analysis, we set *S* to zero (Fig. 4)—the control shows that the *S* parameter is useful, but the conclusions do not depend on it. The model is optimized via gradient descent, using the ADAM optimization method (Kingma and Ba, 2017), and implemented in PyTorch v.1.13.

The SSE method is similar in some respects to LDA. LDA finds a projection hyperplane that maximizes Fisher's criterion, i.e., the ratio of interclass variance to intraclass variance analytically (Xanthopoulos et al., 2013). Our objective function is related but differs in three important respects. First, LDA is primarily a classification method and projects *c* classes of input data into a space with *c* − 1 orthogonal dimensions. Equivalently, this can be thought of as finding *c* − 1 hyperplanes that best separate the classes. Since we solve our objective function numerically and seek the relationships between classes rather than their classification, we specify the dimensionality of the embedding space. Second, to encourage sparsity, we add L1 regularization of discriminant vectors. Third, instead of between-class variance, we maximize the pairwise Euclidean distance between each class centroid. This allows for control over which class pairs are separated by defining a weighting parameter over the pairwise distance matrix.

After optimization, SSE gives us two useful pieces of information. First, it provides the relative locations of the parcels in *E*. Any reliable structure in the arrangement of the parcels is of interest because the objective function itself makes no attempt to enforce any relationship besides being statistically different from each other. Second, it provides weights of the input features. The features are the basis for separating the parcels. These two pieces of information together provide information about the global relationships between all the parcels with respect to the stimulus features. The analysis therefore discovers the extent to which responses to simple color gratings provide functional signatures for visual cortex parcels defined by eccentricity, upper-versus-lower visual field, and retinotopic area.

Estimates of error in the SSE models were established by bootstrapping. The input fMRI dataset was resampled over independent runs 1,000 times, and a separate randomly initialized SSE model was fit on each resampling. For each model, the columns of *T* span a *k*-dimensional vector subspace *E* of the *m*-dimensional feature space. We are interested in the variability of *E*. However, the spanning components *T* may differ between fits despite *E* being near identical since any *E* has infinitely many unique bases. For each run, we want to find a new T* that is comparable across models. In a three-dimensional feature space, *E* would be a plane spanned by two vectors, the columns of *T*. We are interested only in how *E* is angled in this space. For every independent run, we find a rotation matrix in *E* that minimizes the variance over all *T*, yielding T*. Variance over all T* will be nonzero only due to variance in *E* between models. This allows us to ignore random variance in the rotation of the basis and focus on the variance in T* caused by differences in the lower-dimensional representations of the full feature space. We use bootstrapping to estimate the distributions of the model parameters and the mean of the data projection onto *E*. For additional confirmation of model results, we perform cross-validation. The data were divided into two sets composed of even and odd runs. The model was fit (i.e., *T* was estimated) on one dataset and tested (i.e., data were projected onto *E*) on the other independent dataset.

*β* coefficients of the stimuli from Experiments 3 and 4 in both monkeys were concatenated into a voxels × conditions matrix. Each voxel was assigned to a retinotopic area and an eccentricity preference. We ran two SSE analyses that differ in *M*. First, we maximized the separability of different ROIs across the four eccentricity parcels. To do this, we set *M* such as to not consider differences across the different retinotopic areas either within or between eccentricity levels; the only relationships considered were between different eccentricity levels of the same retinotopic area. This allowed us to find one component that best discriminates between more central and more peripheral parcels. Second, we set *M* to ignore differences across eccentricity, only considering the pairwise distance between all retinotopic areas within each eccentricity level. This has the effect of limiting differences between ROIs due to variation in the size of eccentricity representations. The positive and negative features of the recovered components were *z*-score contrasted and projected onto the inflated surface of M1, to show the topography of the response to the different component vectors. In these contrasts, the positive and negative features were scaled to sum to 1 and −1 respectively. Table 3 lists the SSE conditions and their corresponding stimuli, illustrated in Figure 2.

**Table 3.** Conditions corresponding to feature names used in display of SSE discriminant/component vectors

Feature nomenclature	Grating description(s)
LM_high_sat	DKL 0 95% saturation, DKL 180 95% saturation
LM_med_sat	DKL 0 50% saturation, DKL 180 50% saturation
LM_low_sat	DKL 0 30% saturation, DKL 180 30% saturation
LM_xlow_sat	DKL 0 10% saturation, DKL 180 10% saturation
S_high_sat	DKL 90 95% saturation, DKL 270 95% saturation
S_med_sat	DKL 90 50% saturation, DKL 270 50% saturation
S_low_sat	DKL 90 30% saturation, DKL 270 30% saturation
S_xlow_sat	DKL 90 10% saturation, DKL 270 10% saturation
Daylight_high_sat	DKL 45 95% saturation, DKL 225 95% saturation
Daylight_med_sat	DKL 45 50% saturation, DKL 225 50% saturation
Daylight_low_sat	DKL 45 30% saturation, DKL 225 30% saturation
Daylight_xlow_sat	DKL 45 10% saturation, DKL 225 10% saturation
Antidaylight_high_sat	DKL 135 95% saturation, DKL 315 95% saturation
Antidaylight_med_sat	DKL 135 50% saturation, DKL 315 50% saturation
Antidaylight_low_sat	DKL 135 30% saturation, DKL 315 30% saturation
Antidaylight_xlow_sat	DKL 135 10% saturation, DKL 315 10% saturation
LMS_high_sat	LMS 8.7% luminance contrast
LMS_med_sat	LMS 5.5% luminance contrast
LMS_low_sat	LMS 2.5% luminance contrast
LMS_xlow_sat	LMS 1% luminance contrast
LM_high_freq	LM 8 CpD
LM_med_freq	LM 4 CpD
LM_low_freq	LM 0.2 CpD
S_high_freq	S 8 CpD
S_med_freq	S 4 CpD
S_low_freq	S 0.2 CpD
LMS_high_freq	High luminance 8 CpD
LMS_med_freq	High luminance 4 CpD
LMS_low_freq	High luminance 0.2 CpD

### Univariate analysis

For the univariate analyses, *β* coefficients for each condition were calculated for each run in which that stimulus condition was presented. Statistical tests used the *β* coefficients from each run as individual observations. Percentage signal change for each condition was calculated by dividing the *β* coefficient of the condition by the *β* coefficient of the baseline and multiplying by 100.

We computed features that describe the extent to which each voxel was modulated by the LM, S, daylight, and antidaylight conditions using the fMRI responses to Experiment 3. Each condition was estimated by averaging the *β* coefficients computed from the two color-gray gratings that comprise the feature. For example, the LM feature was calculated using responses elicited by the 0° (pink–gray) grating and the response elicited by the 180° (cyan–gray) grating. Pairs of gratings that made up these features always appeared within the same run at the same saturation, and responses to them were averaged for subsequent statistical analysis.

**Figure 3.**
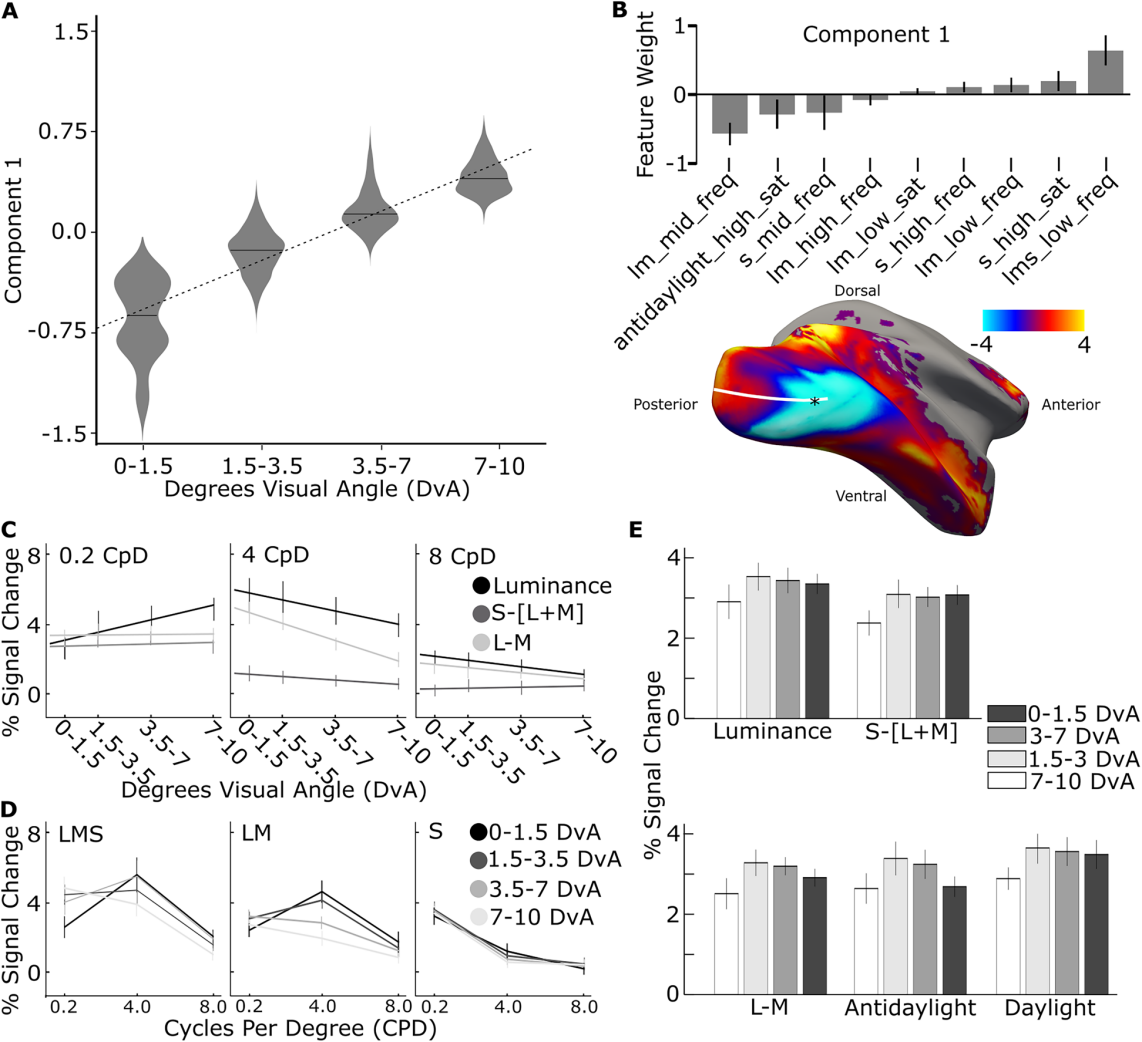
Functional signature of cortical representations of eccentricity recovered with SSE using responses to colored gratings varying in hue, saturation, and spatial frequency. ***A***, One SSE model was fit on all fMRI data (Experiments 3 and 4) obtained in two macaque monkeys. Masking parameter *M* (Eq. 1) was set to constrain the model to separate parcels by the eccentricity preferences of the voxels and not their retinotopic area or hemifield (upper/lower) representation (see Materials and Methods). ***B***, Top, The feature weights for the eccentricity-discriminating component. Only features with weight significantly different from zero are shown. Bottom, Projection of *z*-score contrast between positively and negatively weighted features on M1's cortical surface. ***C***, The response to heterochromatic gratings varying in color (luminance, LM, and S) and spatial frequency (0.2, 4, and 8 CpD), across different eccentricity-preferring parcels. ***D***, Same data as Figure 3*C*, replotted to display frequency response functions for luminance, LM, and S axes at each eccentricity level. ***E***, The response to the highest-saturation color–gray gratings (0.5 CpD) within cortical parcels with different eccentricity preference.

In all plots of different univariate metrics (Figs. 3*C*,*D*, 8, 9), error bars were estimated via bootstrapping over 1,000 iterations. Each iteration's input *β* matrix was obtained by averaging *N* samples drawn with replacement from the set of responses to each feature, where *N* was the total number of stimulus blocks for that feature.

Contrast response functions (CRFs) were generated by plotting the fMRI signal change as a function of the Michelson cone contrast of the stimuli (Fig. 8*A*), computed as the vector length of the individual cone contrasts (Table 1), using the approach of Liu and Wandell (2005), and then averaged across the two monochromatic gratings. This calculation is detailed in Equation 2, where L, M, and S are the individual cone contrasts and the subscripts *a* and *b* indicate the monochromatic grating type.

$$\text{Contrast}=\frac{\sqrt{{\text{L}}_{a}^{2}+{\text{M}}_{a}^{2}+{\text{S}}_{a}^{2}}+\sqrt{{\text{L}}_{b}^{2}+{\text{M}}_{b}^{2}+{\text{S}}_{b}^{2}}}{2}$$

So for LM_high_sat gratings, *a* would be the DKL 0 95% saturation grating, *b* would be the DKL 180 95% saturation grating, and the overall contrast would be 12.0. The CRF slopes and errors were estimated by fitting a linear regression model to the signal change of each feature as a function of cone contrast for each ROI, repeated for each bootstrapped sample (Fig. 8*B*). The statistical tests comparing the slopes were computed using a one-way ANCOVA. The difference in luminance bias for dorsal-versus-ventral parcels was computed by subtracting the bootstrapped luminance response from the bootstrapped color response for both ventral and dorsal regions and then subtracting the dorsal difference from the ventral difference (Fig. 9*B*).

### Surgical procedures

Surgical methods are the same as described in Lafer-Sousa et al. (2012).

## Results

To what extent can retinotopic parcels be distinguished by fMRI responses to simple grating stimuli that vary in spatial frequency, color, and contrast? To answer the question, we collected fMRI responses to these stimuli in two macaque monkeys, and we fit aSSE model to the responses of cortical parcels defined by retinotopic area, upper-versus-lower visual field, and eccentricity preference of each voxel. The SSE model finds a sparse linear transformation (*T*) of the *β* coefficients for each stimulus condition that maximizes the pairwise distance between the mean of each parcel while minimizing variances within each class. As described in the methods, the outcome of the SSE model discovers a low-dimensional stimulus feature space that maximally separates the parcels, and the resulting organization of the parcels is informative about their functional relationships.

### Functional signatures of eccentricity responses

We first used the SSE model to determine a functional signature of eccentricity, irrespective of the retinotopic area. To do this we disregarded the separation of visual area ROIs—setting the indexes in *M* that correspond to pairwise distances between ROIs within the same eccentricity level to zero. The result yields a single component along which the eccentricity parcels are systematically arranged (Fig. 3*A*). The feature contributions to the discriminating component (Fig. 3*B*, top) show that more foveal voxels were defined by higher relative weighting for responses to higher spatial frequency (LM) gratings and to a lesser extent high saturation antidaylight gratings and middle frequency S gratings. More peripheral voxels were characterized by higher relative weighting for responses to low-spatial-frequency (LMS) gratings and high saturation S gratings. Figure 3*B* projects the contrast between positive and negative weighted features of this component on the inflated cortical surface of M1 (Fig. 3*B*, bottom). The result discovers the foveal confluence of the retinotopic cortex (centered on the asterisk, compare with Fig. 1*B*). Foveal representations were also discovered further along the inferior temporal cortex, confirming prior reports using a univariate contrast (Lafer-Sousa and Conway, 2013).

The features that comprise the eccentricity component are consistent with the expectation that foveal representations of the visual cortex are tuned for higher spatial frequencies compared with peripheral representations, in both humans (Henriksson et al., 2009; Broderick et al., 2022) and monkeys (Schiller et al., 1976; De Valois et al., 1982). Peripheral representations, meanwhile, are expected to be more responsive to low spatial frequencies and S cone signals given that the relative proportion of S cones (Curcio et al., 1991) and the apparent saturation of S cone-modulating stimuli (Vanston and Crognale, 2018) increase with eccentricity (pre-retinal macular pigment may also play a role).

The results of the SSE model are multivariate. To compare the results with published reports that use univariate methods, we computed a univariate measure of the responses to the various stimulus conditions as a function of eccentricity. Figure 3*C* shows how responses to the 0.2, 4, and 8 CpD gratings vary as a function of eccentricity, for the three types of gratings defined by the three cardinal directions in cone-opponent color space (luminance, S, and L-M). Responses to low-frequency luminance gratings increased from fovea to periphery [slope, 0.30; *p* = 0.01; *F* test (df, 106)]—this result is consistent with an increase in response to low-spatial-frequency luminance gratings from fovea to periphery observed in the human visual cortex (Bayram et al., 2016). Responses to the higher spatial frequencies (4 and 8 CpD) decreased with eccentricity for both LM and LMS gratings (for 4 CpD, Lum slope, −0.22; *p* = 0.004; S − [L + M] slope, −0.06; *p* = 0.034; L-M slope, −0.35; *p* = 0.007; for 8 CpD, Lum slope, −0.13; *p* = 0.003; S − [L + M] slope, 0.02; *p* = 0.39 L-M slope, −0.11; *p* = 0.035; *F* tests; df, 106). Figure 3*D* provides another view on these data, plotting spatial frequency response functions for LMS, LM, and LMS gratings at each eccentricity level. For comparison, results obtained in humans with fMRI show gradual increases in responses for low-frequency (0.27 CpD) chromatic gratings as one goes from the fovea to the periphery and gradual decreases in responses to higher-frequency (4.4 CpD) L-M, but not S, gratings (D’Souza et al., 2016).

These relative patterns in response are leveraged in the multivariate analysis, which uncovers the combination of features that best perform the separation of fovea from periphery. Because the multivariate analysis is determined by the relative responses among a set of stimuli, it is not always easy to visualize its outcome given univariate analyses, which simply report the magnitude of responses to individual stimuli independent of the responses to any other stimuli. But as the analyses relating to eccentricity show, the outcome of the multivariate analysis is consistent with the univariate analysis.

To begin to relate published perceptual measurements to color, contrast, and spatial frequency, to cortical responses (often reported with univariate analyses), we investigated the univariate responses across eccentricity to the highest-saturation low-spatial-frequency gratings. Figure 3*E* shows the responses to the highest-saturation color–gray gratings (0.5 CpD) for each eccentricity level. Responses to luminance, daylight, and S gratings were lower for the fovea-preferring voxels, while responses to antidaylight and LM gratings were not different for fovea-preferring and periphery-preferring voxels (*t* test, response of foveal-vs-peripheral voxels, luminance *p* = 0.004; S − [L + M], *p* = 0.0006; L-M, *p* = 0.087; antidaylight, *p* = 0.97; daylight, *p* = 0.0016; Tukey's HSD). For comparison, Vanston and Crognale (2018) found that suprathreshold stimuli in the periphery, compared with the fovea, were perceived with lower contrast for LM stimuli and higher contrast for S stimuli. Despite there being no difference in antidaylight high saturation response between the most central and most peripheral parcels, this condition appears significant for separating the eccentricity parcels in the SSE analysis. This is likely because the antidaylight condition elicits clearly different responses for the two intermediate eccentricity parcels.

The univariate and SSE analyses uncover patterns of response largely expected from fMRI in humans. Responses in human V1 elicited by LMS, S − [L + M], and L-M gratings with a spatial frequency of 0.55 CpD increase as one goes from the central 2° to 8–10°  (D’Souza et al., 2016). Luminance gratings cause the greatest increase in response from foveal-preferring voxels compared with peripheral-preferring voxel, while S − [L + M] gratings elicit the smallest increase. Mullen et al. (2007) show that S − [L + M] gratings, also with a spatial frequency of 0.5 CpD, elicit the greatest increase in response from 1°- to 6°-preferring voxels of human V1. They also report a decrease in response elicited by L-M gratings across eccentricity.

The univariate results show an asymmetry in the responses to daylight versus antidaylight colors as a function of eccentricity: daylight colors were better than antidaylight colors at separating fovea from periphery (Fig. 3*E*, bottom panel). As far as we are aware, no data have been published comparing responses to antidaylight and daylight gratings across eccentricity in human subjects. Goddard et al. (2010) did find generally higher fMRI responses to antidaylight gratings than daylight gratings across the visual cortex. The stimuli in that study were somewhat different from those used presently: they were presented on a luminance-contrast pedestal (not equiluminant), were lower mean luminance, increased in spatial frequency from the center to the edge, and were circular.

### Control analyses

**Figure 4.**
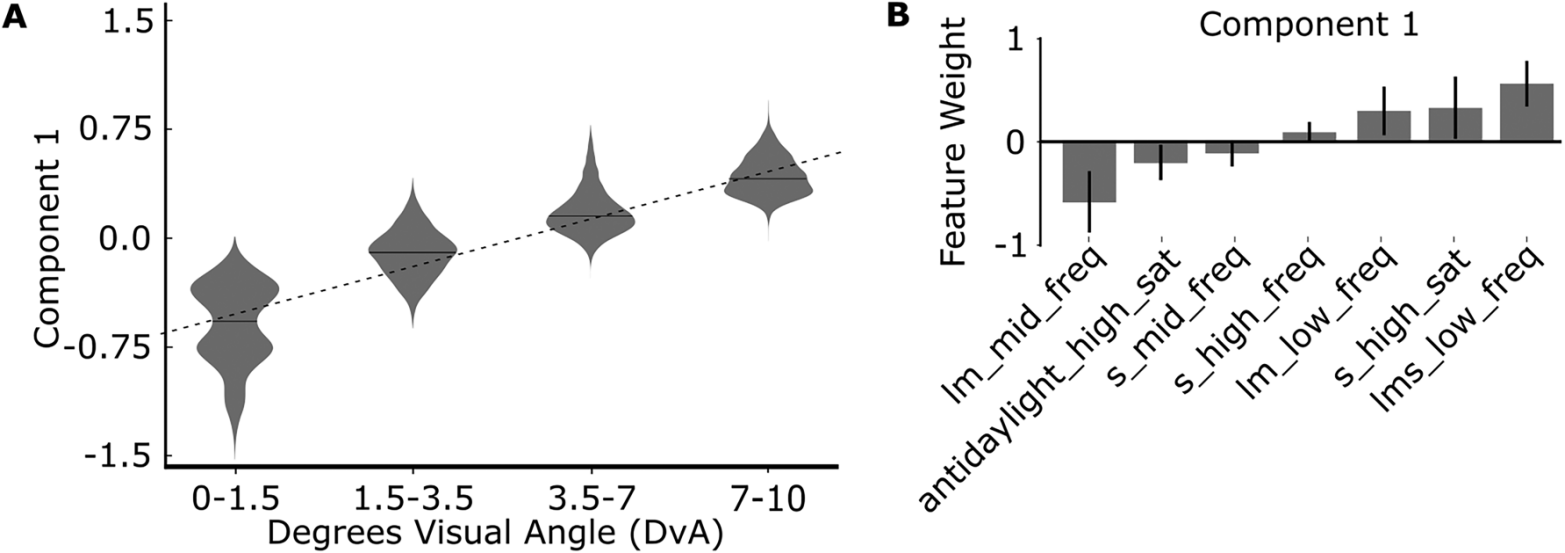
Cortical representations of eccentricity recovered with SSE without L1 regularization, i.e., Sparsity parameter is set to 0. ***A***, One SSE model was fit on all fMRI data (Experiments 3 and 4) obtained in two macaque monkeys. Masking parameter *M* was set to constrain the model to separate parcels by eccentricity preferences of the voxels and not their retinotopic area or hemifield (upper/lower) representation (see Materials and Methods). The response to the component increases with eccentricity (linear regression slope, 0.33; *p* = 0.0). ***B***, The feature weights for the eccentricity-discriminating component. Only features with weight significantly different from zero are shown.

The SSE analysis includes a sparsity parameter, which is a scalar coefficient on an L1 (or Lasso) regularization term in the objective function. This can improve generalization and interpretability, although the specific setting for the sparsity parameter is not well prescribed. To test the impact of the sparsity setting, we performed the eccentricity analysis by setting the sparsity parameter to zero. The results are comparable with those obtained with the sparsity setting of 0.005 (Fig. 4). The six strongest features are the same (compare with Fig. 3). The inclusion of a sparsity value in the analysis recovers two additional components, both having relatively weak contributions. We include the sparsity setting throughout the analysis even though it does not have a substantial impact on the conclusions because it appears to extract more information and may be useful if the SSE approach is adopted for other more complex neuroscience applications.

**Figure 5.**
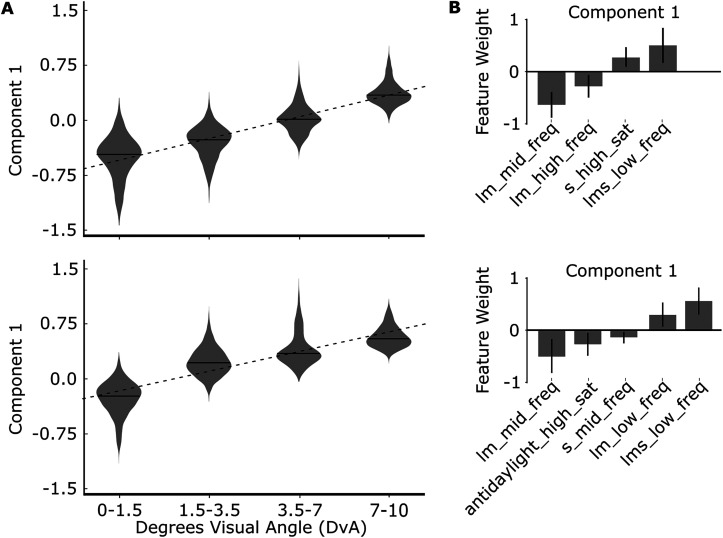
Cortical representations of eccentricity recovered with SSE validated across two independent datasets, A and B, with L1 regularization, i.e., sparsity parameter is set to 0.005. ***A***, One SSE model was fit on all fMRI data in (Experiments 3 and 4) in Set A and the projection of Set B is shown (top), and one was fit on Set B and the projection of Set A is shown (bottom). ***B***, The feature weights for the eccentricity-discriminating component. Only features with weight significantly different from zero are shown.

We also assessed the reliability of the results by cross-validation. We fit (i.e., found component *T*) and evaluated (i.e., projected the parcels onto the subspace spanned by *T*) the model using separate datasets, *A* and *B* (Fig. 5). *A* and *B* were alternate runs of fMRI data collected in Experiments 3 and 4. The cross-validated results show higher variability than the results obtained with all the data due to the decreased power in the subdivided dataset (evident as relatively larger error bars and fewer significant components), but the main conclusions drawn from the analysis are consistent with those obtained using all the data (Fig. 3).

### Functional signatures of retinotopic visual areas

**Figure 6.**
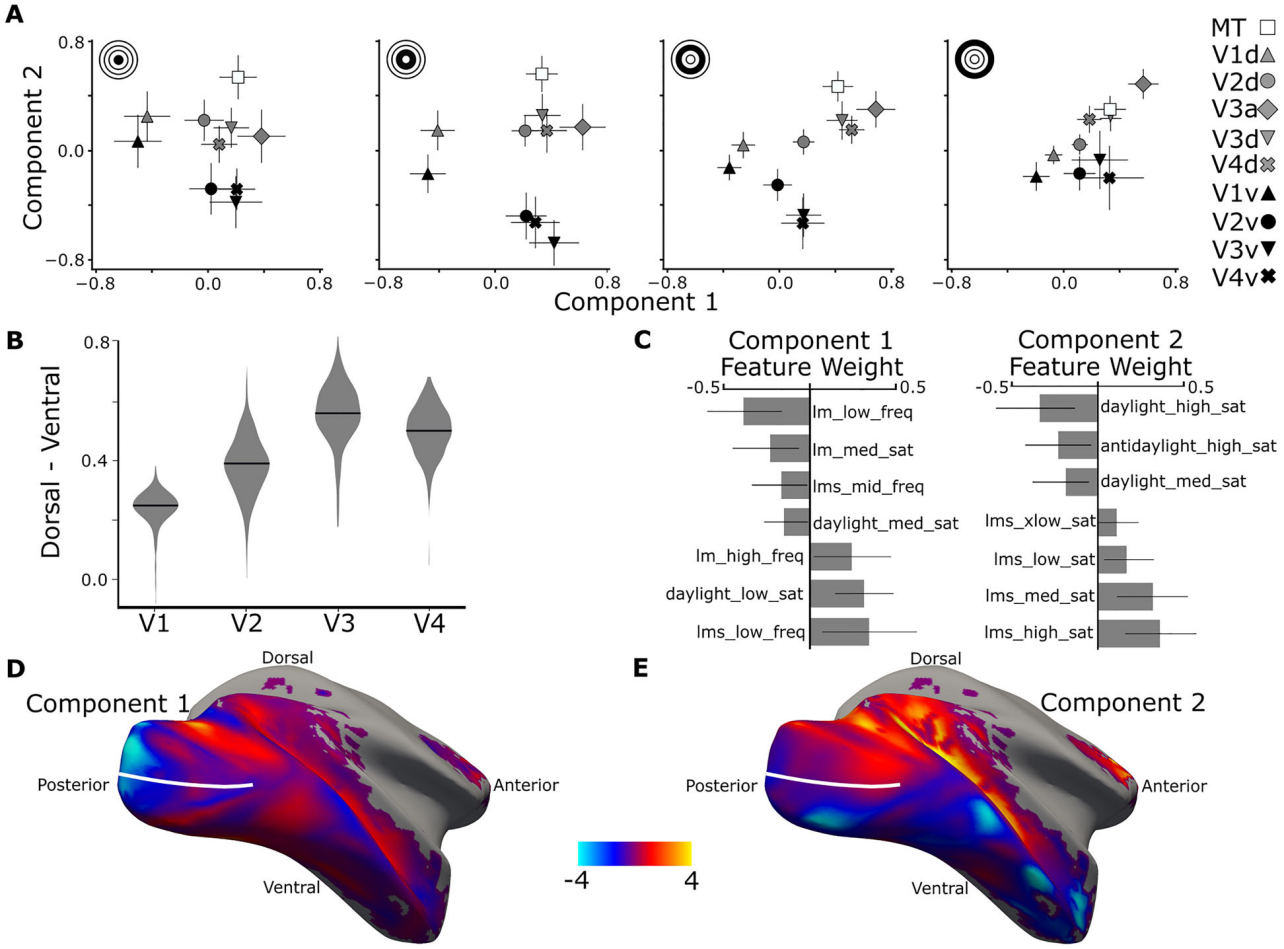
Functional signatures of cortical representations of retinotopic areas and their dorsal/ventral subdivisions recovered with SSE using responses to colored gratings varying in hue, saturation, and spatial frequency. ***A***, The mean voxel response for retinotopic areas and their dorsal/ventral subdivisions along the two maximally discriminating components discovered by the SSE model, for voxels defined by eccentricity preference (inset icon, most foveal-preferring in the leftmost panel; most peripheral-preferring in the rightmost panel. Error bars are 95% confidence intervals via 1,000 bootstrapping iterations). ***B***, The difference in the Component 2 response between dorsal and ventral subdivisions of each retinotopic area. Violin plot distributions estimated by 1,000 bootstrapping iterations. The difference increases along the visual hierarchy. ***C***, The feature contributions of Components 1 and 2. Only features significantly different from zero are shown. ***D***, A surface projection of the *z*-score contrast between the two features in Component 1 with the most positive weights and the most negative weights. ***E***, As in ***D***, a surface projection of the *z*-score contrast between the two features in Component 2 with the most positive weights and most negative weights. The dotted line shows the division between dorsal and ventral parcels.

We next used the SSE approach to test for functional signatures of the retinotopic visual areas and their dorsal-versus-ventral subdivisions. To do this, we fit the SSE model with two components using information matched for each eccentricity preference (Fig. 6), which removes the impact of eccentricity on the voxel responses. The result of this analysis recovers a striking pattern. The first component separated parcels along the putative visual-processing hierarchy, from left-to-right (*x*-axis) in each panel of Figure 6*A*: V1, V2, and V3/V4 (upper triangle, circle, inverted triangle, and cross). The second component separated the ventral and dorsal subdivisions of each visual area, top-to-bottom (*y*-axis): V1d, V2d, V3d, and V4d showed more positive weight compared with V1v, V2v, V3v, and V4v (gray symbols are above black symbols). The second component grouped the two dorsal areas, V3a (diamond) and MT+ (square), with the dorsal subdivisions of the other retinotopic areas.

The difference between ventral and dorsal parcels along Component 2 was progressively greater along the putative visual-processing hierarchy (Fig. 6*B*; slope, 0.09; *p* = 1.1 × 10^−98^). A dorsal/ventral asymmetry in V1 has not previously been described; we note that it was only evident in the present report using the multivariate analysis which recovers structure across visual areas and eccentricities. The value for Component 2 was only significantly different for dorsal and ventral V1 parcels for the three peripheral parcels (Fig. 6*A*).

Figure 6*C* shows the feature contributions for the two components. Component 1 corresponds to strong differences between negative-weight features (lm_low_freq, lm_med_sat, lms_mid_freq, and daylight_med_sat) versus positive-weight features (lm_high_freq, dalight_low_sat, lms_low_sat). The sets of positive-weight features and negative-weight features are each not explained by a simple common feature property. For example, the negative-weight features include stimuli with relatively higher saturation (the lm_low_freq stimulus is a heterochromatic grating with reddish and greenish bands that has the highest color contrast of any stimulus we used) as well as stimuli that have no color contrast (e.g., the lms_mid_freq stimulus), while the positive-weight features include stimuli with low and high spatial frequency. The positive-weight features are low saturation or low color contrast, except the lm_high_freq stimulus which has high saturation but also high spatial frequency. The negative-weight features are high color contrast or high saturation and relatively lower spatial frequency compared with the positive-weight features. Component 2 predominantly reflects differences in responses to color versus luminance, particularly responses to daylight-axis colors. We underscore that these analyses do not recover the univariate features that most strongly drive voxels but rather uncover the sets of relative differences (reflecting interactions among features) that capture the functional signatures of the voxels. These complex functional signatures can be explored by investigating univariate responses, as we do in subsequent analyses (Figs. 8, 9).

To help visualize the results, we projected on the cortical surface of the right hemisphere of M1 the contrast map generated by contrasting positive-weight features with negative-weight features, for Component 1 (Fig. 6*D*) and Component 2 (Fig. 6*E*). The surface projection of Component 1 shows a gradient progressing from posterior (bluer) to anterior (redder), whereas the surface projection of Component 2 is predominantly redder in dorsal parcels (above the white line) and bluer in ventral parcels (below the white line).

**Figure 7.**
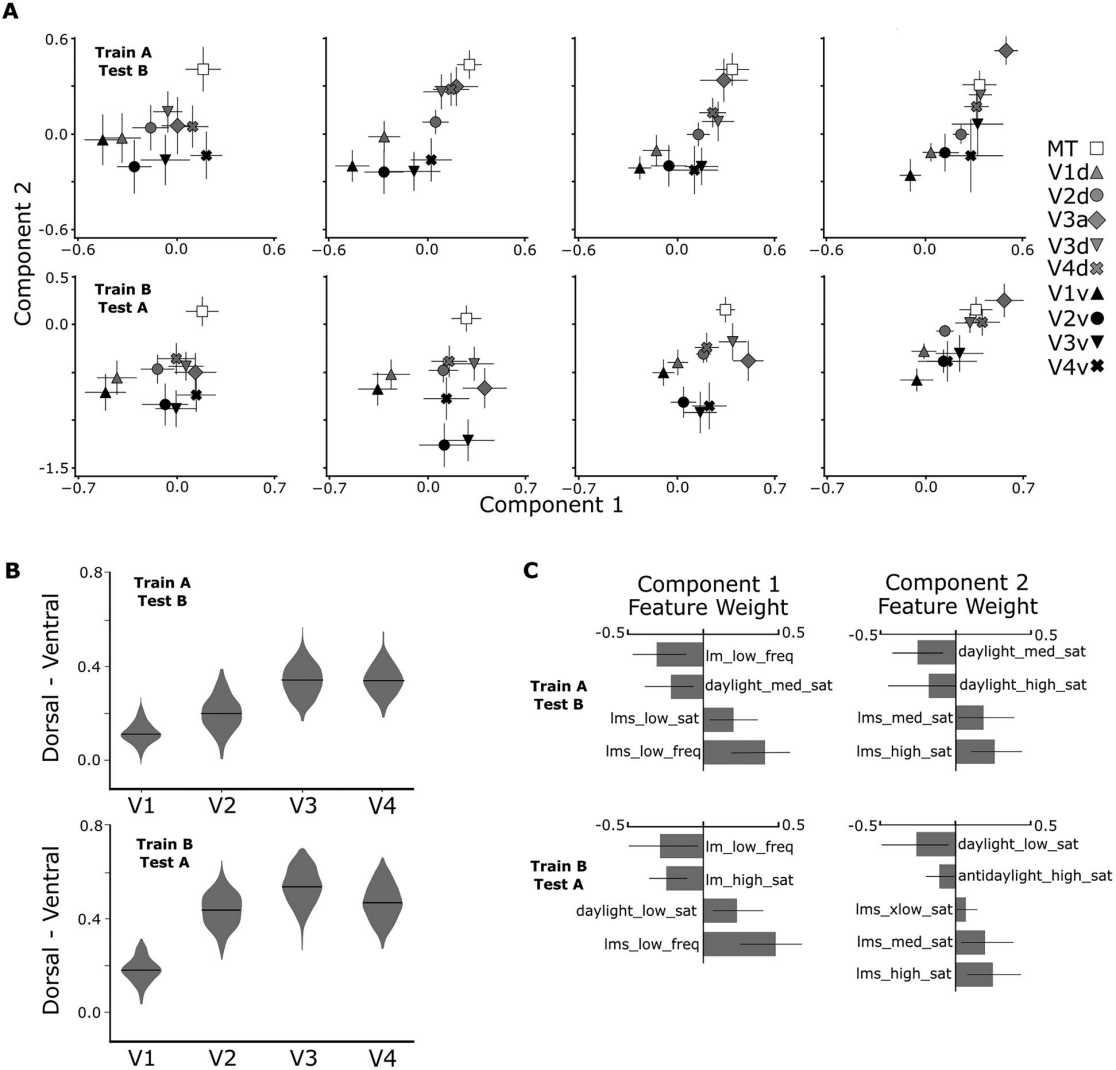
Functional signatures of retinotopic areas and their dorsal/ventral subdivisions recovered with SSE validated across two independent datasets, A and B, with L1 regularization, i.e., sparsity parameter is set to 0.005. ***A***, One SSE model was fit on all fMRI data in (Experiments 3 and 4) in Set A and the projection of Set B for each eccentricity level is shown (top), and one was fit on Set B and the projection of Set A for each eccentricity level is shown (bottom). ***B***, The difference in the Component 2 response between dorsal and ventral subdivisions of each retinotopic area. Violin plot distributions estimated by 1,000 bootstrapping iterations. The difference increases along the visual hierarchy (top, slope, 0.069; *p* = 1 × 10^−26^; bottom, slope, 0.0823; *p* = 1 × 10^−31^). ***C***, The feature weights for the eccentricity-discriminating components fit on Set A (top) and Set B (bottom). Only features with weight significantly different from zero are shown.

We validate the SSE results by cross-validation: fitting and evaluating on separate independent datasets, *A* and *B*, composed of alternating runs of fMRI Experiments 3 and 4 (Fig. 7). As in the earlier cross-validation, variability increases due to the decreased power in the subdivided dataset (evident as relatively larger error bars and fewer significant components), but the main conclusions are consistent with those obtained using all the data (compare Fig. 7 with Fig. 6).

To better understand what underlies the functional signatures of visual areas and their dorsal/ventral subdivisions, we related the outcomes of the SSE analyses and the univariate analyses. But once again, we underscore that the SSE recovers interactions among features that constitute the functional signatures not a straightforward combination of univariate features. The output of the SSE analysis might not be easily visualized as combinations of univariate results, even though we believe it provides important clues regarding the computational role(s) of the cortical parcels.

**Figure 8.**
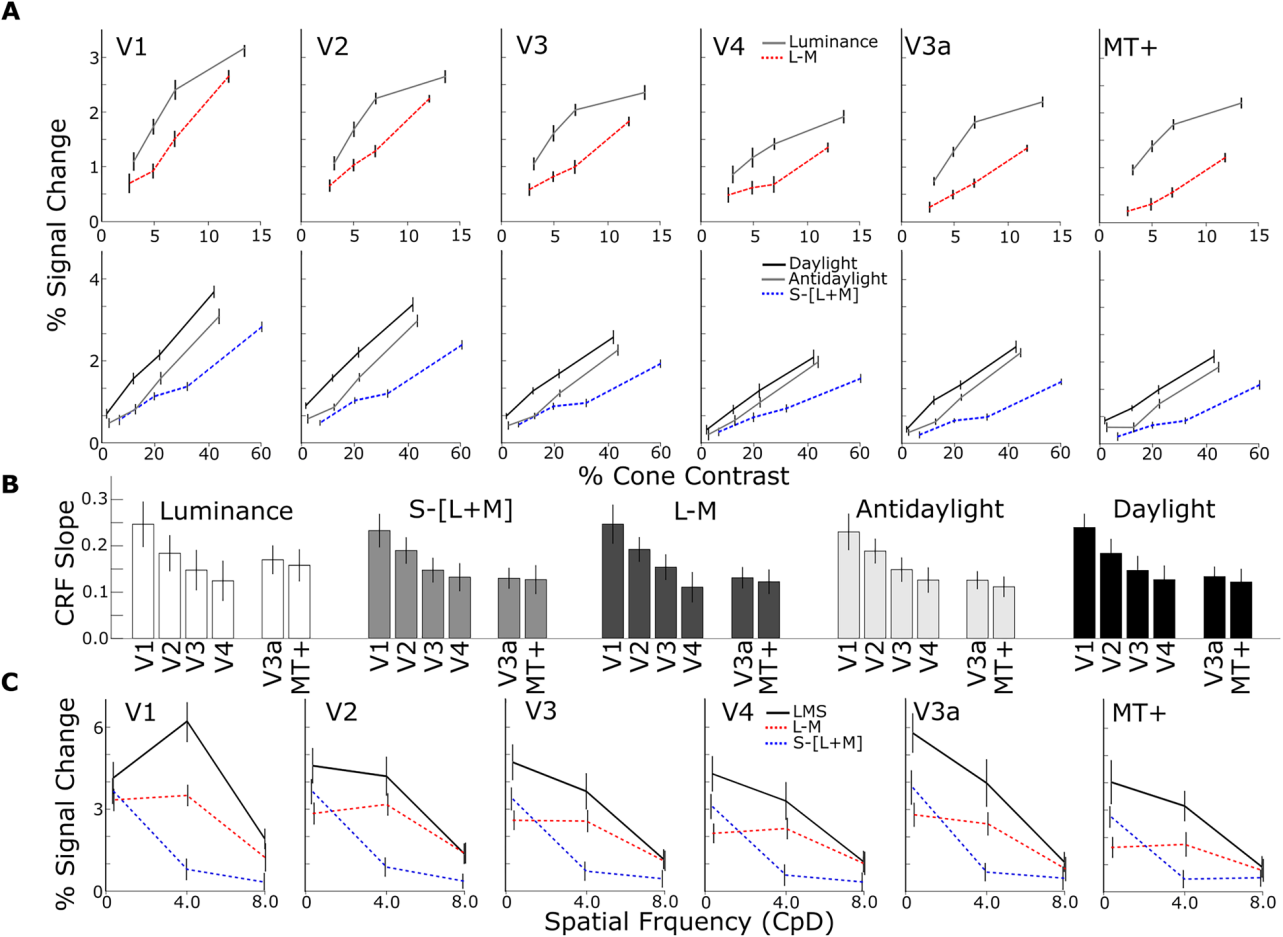
Univariate analyses of the responses of retinotopically defined parcels to colored gratings varying in saturation or spatial frequency. ***A***, CRFs for LM, S, luminance, daylight, and antidaylight gratings in each ROI. Cones contrast is the average Michelson cone contrast of the two monochromatic gratings on each axis in DKL color space. ***B***, CRF slope for the fMRI responses to colors defined by various directions through color space (top labels) decrease from V1 to V4 (error bars represent 95% C.I.); CRFs for V3a and MT are shown on the right. The CRF is progressively shallower for areas from posterior to anterior. ***C***, Spatial frequency response functions for LM, S, and LMS gratings in each ROI.

**Figure 9.**
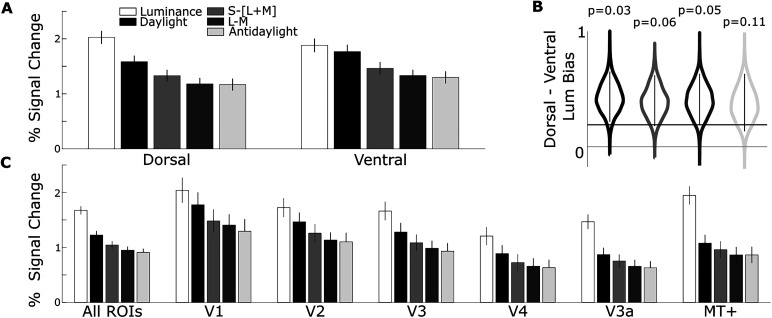
***A***, The percentage fMRI signal change in response to the gratings defined by various directions through color space (key) for the dorsal and ventral subdivisions of V1, V2, V3, and V4. Each bar shows the average response to the three gratings of highest saturation for each color axis (Fig. 3*C*). The responses to gratings modulated along the luminance axis were significantly greater than the responses to gratings modulated along the daylight axis in dorsal parcels, but not ventral parcels (two-way ANOVA, main effects of color, *F* = 58; *p* = 0; region, *F* = 6.3; *p* = 0.01; and an interaction of region and color, *F* = 2.7; *p* = 0.03; post hoc test comparing response to luminance gratings vs daylight gratings, Tukey's HSD, dorsal luminance–dorsal daylight, *p* = 2.8 × 10^−6^; ventral luminance–ventral daylight, *p* = 0.93). ***B***, Violin plot comparing dorsal-versus-ventral regions for the difference between the responses to gratings modulating along the luminance axis versus the responses to gratings modulating along the other color axes (from left to right, daylight, S, L-M, and antidaylight). Dorsal parcels show a bias for luminance regardless of what color is used as the comparative response (note the *p* values in the plot), but the bias is greatest when compared with responses along the daylight axis; violin plot distributions estimated by 1,000 bootstrap iterations. ***C***, The percentage signal change for gratings defined by each direction through color space, combined across all saturation levels. Gratings along the daylight axis elicited a greater response than colored gratings along all other axes (excluding luminance) for responses averaged across all retinotopic parcels (left plot, daylight vs S, *p* = 0.001; daylight vs LM, *p* = 8.9 × 10^−10^; daylight vs antidaylight, *p* = 2.9 × 10^−10^). Right panels show this analysis for each visual area separately (multiple comparisons render statistical tests insignificant). Error bars are 95% confidence intervals computed by bootstrapping across runs.

In the SSE analysis that separated parcels by the visual area (Fig. 6*C*, Component 1), negative-weight features correspond to more posterior areas and positive-weight features correspond to more anterior areas. Component 1 in that analysis was defined by one strong positive-weight feature that has no chromatic contrast, while the two strongest negative-weight features have relatively high chromatic contrast. This pattern of results suggests that differences in chromatic CRFs are partially what distinguish posterior from anterior visual areas. We analyzed the fMRI responses to the different saturation stimuli as a function of visual area and dorsal/ventral subdivision (Figs. 8, 9). Figure 8*A* shows the CRFs for each visual area to each color axis and luminance gratings. We determined the slope of the CRFs for stimuli defined by each color axis within each visual area (slopes were a line fit to the fMRI response as a function of contrast; see Materials and Methods; Fig. 8*B*). There was a main effect of parcel on the CRF slope; CRFs for all color axes were progressively shallower from posterior to anterior visual areas.

Component 1 also recovers significant weight for the features corresponding to low-spatial-frequency luminance and LM gratings. We show spatial frequency response functions in Figure 8*C*. Response to low-spatial-frequency luminance gratings was relatively lower in V1 compared with more anterior areas, and response to lower frequency LM gratings was relatively higher in V1 compared with more anterior areas. Note that the difference between low-spatial-frequency LMS and LM gratings increases along the visual hierarchy, a feature that is uncovered by the SSE analysis in Component 1 (Fig. 6*C*).

The SSE model also suggests that differential responses to daylight and luminance gratings separate dorsal and ventral parcels. To evaluate this directly, we plot the fMRI responses to each color grating averaged across the four saturation levels for dorsal-versus-ventral parcels (Fig. 9*A*). There was a difference in fMRI responses of dorsal and ventral parcels [two-way ANOVA, main effects of color (*F* = 58; *p* = 0), region (*F* = 6.3; *p* = 0.01), and to a lesser extent an interaction of parcel and color (*F* = 2.7; *p* = 0.03)]. Post hoc tests reveal that although responses to individual color conditions did not differ between dorsal and ventral parcels (Tukey's HSD, *p* > 0.05), responses to luminance-contrast gratings were greater than responses to daylight gratings for dorsal but not ventral parcels (Tukey's HSD, dorsal luminance–dorsal daylight, *p* = 2.8 × 10^−6^; ventral luminance–ventral daylight, *p* = 0.93).

To investigate the increase in color response relative to luminance response in the ventral subdivisions compared with the dorsal subdivisions, we computed a univariate feature specified by the SSE results. The feature is the difference in luminance bias between dorsal and ventral parcels, where “luminance bias” was computed as the difference in response to luminance gratings and the response to each of the different colored gratings. The results are shown in Figure 9. The dorsal parcels showed a luminance bias regardless of which color grating was used to assess it, although not all the comparisons were significant (daylight-vs-luminance, *p* = 0.03; LM-vs-luminance, *p* = 0.05; S-vs-luminance, *p* = 0.06; antidaylight-vs-luminance, *p* = 0.11; one-sided bootstrap *t* tests; Fig. 9*B*).

The signatures of retinotopic areas and their dorsal/ventral subdivisions recovered by SSE consistently assigned high weight to the responses to daylight-axis modulating gratings (daylight-axis gratings are weighted highly in both Components 1 and 2; Fig. 6*C*). So, we investigated whether cortical responses were higher to gratings that modulated along the daylight axis compared with gratings that modulated along other directions in color space. Across all retinotopic parcels, the average fMRI response was highest to luminance-modulating gratings (Fig. 9*C*, open bars). Among the responses to colored gratings, the highest response was to gratings that modulate along the daylight axis (Fig. 9*C*, left panel; daylight vs S, *p* = 0.001; daylight vs LM, *p* = 9 × 10^−10^; daylight vs antidaylight, *p* = 3 × 10^−10^). This pattern was consistent in all visual areas (Fig. 9*C*, right panels) but varied somewhat across areas (two-way ANOVA, main effects of both area and color axis; *F* = 85; *p* = 0.0 for ROI; *F* = 61; *p* = 0.8 for color axis). Figure 9*C* also shows the strong luminance bias of V3a and MT as reported previously (Conway and Tsao, 2005).

## Discussion

This study presents fMRI data of the macaque visual cortex to a battery of gratings varying in hue, contrast, and spatial frequency. The data are analyzed both with traditional univariate methods such as contrast-sensitivity curves and with a multivariate dimensionality-reduction approach that we call SSE. Our goal was to discover functional signatures for retinotopic visual areas and their organization by eccentricity and upper/lower visual hemifield. In the SSE analysis, each stimulus condition constitutes one dimension in a high-dimensional space; we used SSE to recover three axes, or components, through this space, each defined by a multivariate combination of stimulus features. One component explained the eccentricity representation; the other two components separated visual areas and their dorsal/ventral subdivisions. The results offer insight into the roles of different visual areas, the origin of behavioral asymmetries between upper and lower visual fields, and the functional similarity between macaques and humans. More broadly, the results provide a proof-of-principle of the utility of the SSE approach in neuroscience.

### A functional signature of cortical responses defined by eccentricity

To recover components unrelated to eccentricity, we set the SSE model to disregard the distance between visual areas and their ventral and dorsal subdivisions. The result is a single component that robustly separates the representation of fovea from periphery (Fig. 3). The features of the component are consistent with expectations: foveal representations are tuned for higher spatial frequencies compared with peripheral representations (Schiller et al., 1976; De Valois et al., 1982; Henriksson et al., 2009; Broderick et al., 2022), and peripheral representations are relatively more responsive to S cone signals (Curcio et al., 1991; Vanston and Crognale, 2018). The result implies that across individuals, the pattern of responses across retinotopic cortex is predictive of stimulus color. In other words, the brain response to a given color is somewhat comparable from one person to the next, as predicted from other neurophysiological measurements (Rosenthal et al., 2021). 

### Functional signatures of retinotopic area and upper/lower visual field representations

To recover components unrelated to eccentricity, we set the SSE model to disregard the separation of parcels with different eccentricity preference. The result was striking: the model discovered two components that discriminated visual areas by their anatomical location and their dorsal/ventral subdivisions (Fig. 6). Components recovered in the SSE analysis correspond to multivariate combinations of the stimuli, which likely reflect the fact that visual neurons are sensitive along multiple dimensions. For example, parvocellular LGN cells, which provide a major input to the visual cortex, show sharper spatial frequency tuning when tested using luminance contrast than color (Wiesel and Hubel, 1966). The functional signature of Component 1, which distinguished posterior areas from anterior areas also reflects the flatter CRFs observed in more anterior areas (Boynton et al., 1996; Olman et al., 2004; Buracas and Boynton, 2007; Tregillus et al., 2021). The functional signature of Component 2, meanwhile, discriminated dorsal parcels from ventral parcels, with strong weight to chromatic features, especially daylight colors. This result extends to macaques an observation in humans: compared with dorsal partitions, ventral partitions across cortical areas generally show relatively greater responses to color (Goddard et al., 2011).

Dorsal V1 has relatively more cortical real estate compared with ventral V1, in monkeys (Van Essen et al., 1984) and humans (Silva et al., 2018). This asymmetry correlates with higher contrast sensitivity in the lower visual field (Himmelberg et al., 2023; recall dorsal V1 maps the ventral visual field). Component 2 here confirms the higher contrast sensitivity among dorsal parcels. But Component 2 cannot be solely explained by higher contrast sensitivity of dorsal parcels, as its features were also related to color modulation. Two Component 2 features comprise daylight-axis colors. Because the color statistics of objects correspond largely to daylight-axis colors (Rosenthal et al., 2018), we speculate that the present results reflect specializations not only of dorsal parcels for higher contrast sensitivity but also ventral parcels for better color detection of behaviorally relevant parts of scenes (objects).

Asymmetries in color versus luminance responses of dorsal and ventral subdivisions of macaque V4 have not been found by others (Wade et al., 2008). The present results suggest that measuring colors defined by intermediate axes, along with controls for eccentricity, may be necessary to make this observation. The present results also suggest that dorsal parcels are separated from ventral parcels for all visual areas, but to a progressively greater extent along the posterior-to-anterior sequence of visual areas, such that the dorsal/ventral asymmetry in V1 is only evident in the multivariate pattern of results not the univariate analyses. The multivariate pattern of results described presently suggest that the asymmetry in dorsal and ventral V4 may be inherited and amplified from V1, just as the foveal bias of V1 is amplified by V4 (Motter, 2009).

### SSE as a probe of monkey–human cortical homologies

To what extent are macaque and human visual areas homologous? Homology is reasonably well supported for V1, V2, V3, and inferior temporal areas (Lafer-Sousa et al., 2016). But there are conflicting views regarding V4 (Wade et al., 2002; Fize et al., 2003; Brewer et al., 2005; Larsson and Heeger, 2006; Hansen et al., 2007; Winawer et al., 2010; Roe et al., 2012; Vanduffel et al., 2014; Winawer and Witthoft, 2015). The argument for species divergence of V4 has taken two routes. The first concerns the retinotopic organization of the dorsal and ventral candidate parcels (Tootell, 2001; Wade et al., 2002; Brewer et al., 2005; Larsson and Heeger, 2006; Hansen et al., 2007; Winawer et al., 2010; Winawer and Witthoft, 2015). The second concerns the extent of color sensitivity. In humans, candidate dorsal V4, to the extent it is identified, shows relatively lower responsivity to color compared with ventral V4, while both dorsal and ventral portions of V4 in monkeys supposedly show comparable responses to color (Goddard et al., 2011). But here we find that dorsal and ventral partitions of monkey V4 diverge in color responsivity, with dorsal regions less responsive to color than ventral regions. While the present results do not resolve whether V4 is the same in macaques and humans, they are consistent with V4 homology across macaques and humans, and they underscore the utility of multivariate tools such as SSE in addressing the question.

### The origin of dorsal/ventral functional asymmetries

Could a dorsal/ventral asymmetry be inherited from asymmetric retinal photoreceptor distributions? The inferior retina has slightly higher cone density than the superior retina in both humans (Curcio et al., 1991; Song et al., 2011) and macaques (Perry and Cowey, 1985; Packer et al., 1999), consistent with the higher chromatic responses of the ventral retinotopic parcels. The superior retina also has relatively more rods compared with the inferior retina, resulting in a higher relative number of rods to cones (Curcio and Allen, 1990). This asymmetry may contribute to the contrast response differences observed between dorsal and ventral parcels. But retinal factors cannot fully explain the cortical asymmetries for at least three reasons. First, the retinal ganglion cell density is markedly higher in the superior retina (Curcio and Allen, 1990; Watson, 2014), opposite to the asymmetry in cone density. Second, cone distributions and preretinal optical factors are insufficient to explain asymmetries in visual performance (Kupers et al., 2019). And third, differences in cone density cannot account for functional asymmetries between dorsal and ventral parcels observed for daylight versus antidaylight colors. We hypothesize that the dorsal/ventral asymmetries across the retinotopic cortex arise from selective sampling of geniculate input by V1, which is then amplified along the cortical hierarchy.

Just as the retinotopic organization of the cortex determines aspects of visual behavior, we expect that the asymmetry of dorsal-versus-ventral parcels also corresponds to computational objectives of the cortex. Responses to daylight colors appear to distinguish ventral and dorsal parcels, particularly in V3 and V4 (Fig. 9*A*). One possibility is that this dorsal/ventral asymmetry reflects an adaptation for processing differences in natural scene statistics between the upper and lower visual field. The dorsal/ventral asymmetry implies that visual discrimination is more likely to have useful information defined by daylight colors in the upper visual field compared with the lower visual field. Daylight-axis colors are important for object recognition and scene segmentation (Lafer-Sousa et al., 2012; Pearce et al., 2014; Rosenthal et al., 2018). A potential behavioral advantage for color-in-service-of-object vision in the upper visual field may parallel the behavioral advantage in the upper visual field found for face perception (Quek and Finkbeiner, 2014, 2015; Tsurumi et al., 2022) and shape perception (Zito et al., 2016).

### Broader applications of the SSE method

Neural tuning properties differentiate retinotopic areas, as discovered using parametrically generated texture stimuli (Freeman et al., 2013; Ziemba et al., 2016). Building on the conceptual framework established by Hubel and Wiesel, which considers complex stimulus selectivity as a chain of computations starting with punctate center-surround receptive fields of retinal ganglion cells, our work uses SSE to determine whether visual areas, not just neurons, can be characterized by functional signatures constructed from simpler building blocks. The present results serve as a proof-of-principle that SSE could be fruitfully applied to identify functional signatures using building blocks beyond gratings. For instance, we aim to use this approach to identify abstract rules common to various subregions (color-biased domains, face patches, place-biased domains) within each of the four stages of the inferior temporal cortex.
